# Retrospective evaluation of clinical decision support for within-laboratory optimization of SARS-CoV-2 NAAT workflow

**DOI:** 10.1128/jcm.00785-23

**Published:** 2023-12-22

**Authors:** Thomas J. S. Durant, David R. Peaper

**Affiliations:** 1 Department of Laboratory Medicine, Yale School of Medicine, New Haven, Connecticut, USA; 2 Biomedical Informatics and Data Science, Yale School of Medicine, New Haven, Connecticut, USA; Boston Children's Hospital, Boston, Massachusetts, USA

**Keywords:** informatics, clinical decision support, ask at order entry questions, computerized provider order entry, turnaround time, SARS-CoV-2, COVID-19, business intelligence

## Abstract

The unprecedented demand for severe acute respiratory syndrome coronavirus 2 (SARS‑CoV‑2) testing led to challenges in prioritizing and processing specimens efficiently. We describe and evaluate a novel workflow using provider- and patient-facing ask at order entry (AOE) questions to generate distinctive icons on specimen labels for within-laboratory clinical decision support (CDS) for specimen triaging. A multidisciplinary committee established target turnaround times (TATs) for SARS-CoV-2 nucleic acid amplification test (NAAT) based on common clinical scenarios. A set of AOE questions was used to collect relevant clinical information that prompted icon generation for triaging SARS-CoV-2 NAAT specimens. We assessed the collect-to-verify TATs among relevant clinical scenarios. Our study included a total of 1,385,813 SARS-CoV-2 NAAT conducted from March 2020 to June 2022. Most testing met the TAT targets established by institutional committees, but deviations from target TATs occurred during periods of high demand and supply shortages. Median TATs for emergency department (ED) and inpatient specimens and ambulatory pre-procedure populations were stable over the pandemic. However, healthcare worker and other ambulatory test TATs varied substantially, depending on testing volume and community transmission rates. Median TAT significantly differed throughout the pandemic for ED and inpatient clinical scenarios, and there were significant differences in TAT among label icon-signified ambulatory clinical scenarios. We describe a novel approach to CDS for triaging specimens within the laboratory. The use of CDS tools could help clinical laboratories prioritize and process specimens efficiently, especially during times of high demand. Further studies are needed to evaluate the impact of our CDS tool on overall laboratory efficiency and patient outcomes.

**IMPORTANCE:**

We describe a novel approach to clinical decision support (CDS) for triaging specimens within the clinical laboratory for severe acute respiratory syndrome coronavirus 2 (SARS‑CoV‑2) nucleic acid amplification tests (NAAT). The use of our CDS tool could help clinical laboratories prioritize and process specimens efficiently, especially during times of high demand. There were significant differences in the turnaround time for specimens differentiated by icons on specimen labels. Further studies are needed to evaluate the impact of our CDS tool on overall laboratory efficiency and patient outcomes.

## INTRODUCTION

Clinical laboratories faced numerous challenges during the severe acute respiratory syndrome coronavirus 2 (SARS‑CoV‑2) pandemic related to the demands for testing and the need to rapidly adapt to new testing methods and technologies (1, 2). Most health systems and laboratories were also strained by staffing shortages, supply chain disruptions, and novel data management requirements (3). As a result, many laboratories had to rapidly adjust operational strategies to meet the demands of the crisis. The surge in testing demand and turnaround time (TAT) requirements for diverse clinical scenarios required some laboratories to significantly change how specimens were triaged and processed.

Specimen triaging is an essential part of the total testing process in clinical laboratories encompassing receiving, sorting, and prioritizing patient specimens submitted (4). Appropriate triaging ensures that specimens are processed in a timely and accurate manner and results are reported back to the patient and healthcare providers promptly. During the pandemic, health care organizations identified clinical scenarios in which a rapid TAT (e.g., <2 hours) was important for patient care, but other clinical scenarios had less urgent TAT requirements. Accordingly, laboratories were often asked to provide SARS-CoV-2 nucleic acid amplification tests (NAAT) within specific TAT target thresholds for different clinical scenarios.

At our institution, TAT targets for different clinical scenarios were developed by a multidisciplinary committee that sought to balance clinical need and laboratory resources, recognizing that providing rapid testing to all patients was not feasible. Over the course of the pandemic, various clinical scenarios (e.g., patient to be admitted to ICU, pre-procedure, and asymptomatic ambulatory testing) were defined with associated TAT targets ranging from 2 to 72 hours.

During the early stages of the pandemic, a review of workflow processes revealed inefficiencies in the laboratory specimen triaging system, making it challenging for laboratory staff to promptly direct specimens to appropriate testing pathways and meet diverse TAT requirements. In addition, there were no easily accessible data points within our LIS that allowed accurate identification and tracking of specimens belonging to each clinical scenario; therefore, there was no way to measure TAT for these groups for quality assurance purposes. Accordingly, process redesign of specimen triaging workflows was necessary to meet institutional TAT targets for specific clinical testing scenarios.

Computerized order entry (CPOE) is a common workflow tool with electronic health record (EHR) systems. CPOE enable healthcare professionals to submit orders for clinical services, including laboratory testing, and ask-at-order-entry (AOE) questions are a specific component of CPOE that contain discrete data entry fields which prompt users to enter specific information related to orders (5, 6). AOE questions can optimize clinical decision support (CDS) tools by optimizing workflows, automating tasks, and reducing the risk of errors and adverse events (7 – 11). Informatics tools such as AOE questions may enable conditional processes that selectively streamline workflows where shorter TATs are needed.

In this paper, we describe the use of AOE questions to drive downstream logic that created unique specimen label icons, corresponding to committee-defined clinical scenarios that were subsequently used by laboratory staff for within-laboratory CDS for specimen triaging. We retrospectively review the effect label icon-based within-laboratory CDS had on compliance with institutional TAT targets.

## MATERIALS AND METHODS

### Sample collection and inclusion criteria

We included all specimens collected and/or tested under the umbrella of Yale New Haven Hospital (YNHH) between March 15, 2020, and April 2, 2022. This encompassed samples collected from the following settings: (1) inpatient and emergency department (ED) units from all YNHH campuses (2), specimens dispatched to YNHH laboratories from affiliated outpatient clinics (3), samples collected at draw stations and ambulatory testing sites managed by YNHH (4), samples sent to YNHH from other hospitals within our health system (internal reference testing), and (5) specimens collected under specific contractual agreements. Included testing was performed at the YNHH main laboratory, at a second inpatient campus, and at a standalone ED.

### Testing procedures and modification protocols

Testing methods used during the study period are summarized in Table 1. All testing methods were performed according to the manufacturers’ guidelines with the exception of the TaqPath COVID-19 Combo Kit, for which we incorporated two modifications. Firstly, we used Ct value thresholds to guide repeat testing to account for potential false-positive results. The Ct value thresholds and repeat criteria varied over the testing period but were typically in the mid- to upper-30s. Secondly, a sample pooling strategy was implemented and intermittently used depending on volume and positivity rates with a 4:1 ratio from March 2021 to September 2021 for the selected samples. Most aspects of testing with the TaqPath COVID-19 Combo Kit were automated using specimen handling robots with procedures consistent with the manufacturer’s instructions except as noted above.

**TABLE 1:** Testing methods used throughout the study period with FDA reference and manufacturer information

Assay name	EUA/DEN	Address
Xpert Xpress CoV-2/Flu/RSV plus	EUA210505	Cepheid 904 Caribbean Drive Sunnyvale, CA 94089
Xpert Xpress SARS-CoV-2	EUA200035	Cepheid 904 Caribbean Drive Sunnyvale, CA 94089
Xpert Xpress CoV-2 plus	EUA220187	Cepheid 904 Caribbean Drive Sunnyvale, CA 94089
SARS-CoV-2 PCR test (YNHH CDC Assay)	EUA200061	Yale New Haven Hospital Clinical Virology Laboratory PS619 20 York Street, New Haven, CT 06504 US
Simplexa COVID-19 Direct	EUA200026	DiaSorin Molecular LLC 11331 Valley View Street, Cyress, CA 90630 US
Aptima SARS-CoV-2 assay	EUA200734	Hologic, Inc. 10210 Genetic Center Drive San Diego, CA 92121
cobas SARS-CoV-2	EUA200009	Roche Molecular Systems, Inc. 4300 Hacienda Drive Pleasanton, CA 94588
cobas SARS-CoV-2 & Influenza A/B	EUA202635	Roche Molecular Systems, Inc. 4300 Hacienda Drive Pleasanton, California 94588–0900
TaqPath COVID-19 Combo Kit	EUA200010	ThermoFisher Scientific, Inc. 5791 Van Allen Way Carlsbad, CA 94080
BioFire Respiratory Panel 2.1 (RP2.1)	DEN200031	Biofire Diagnostics, LLC515 Colorow Drive Salt Lake City, Utah 84108
BioGX SARS-CoV-2 Reagents for BD Max System	EUA200098	Becton, Dickinson & Company (BD) 7 Loveton Circle, Sparks, MD 21152

### COVID-19 testing stewardship committee: clinical scenarios and TAT targets

A multidisciplinary committee spanning across our integrated delivery network (six hospitals) was established in March 2020 to determine Coronavirus disease 2019 (COVID-19) testing priorities, manage the allocation of testing resources among various clinical populations, and define and reassess TAT targets for clinical scenarios (Table 2). Clinical scenarios were defined for ED and inpatient admission and triage testing. Additional clinical scenarios were defined for ambulatory testing requirements for pre-procedure testing, HCW, and symptomatic and asymptomatic community members seeking testing. The committee operated continuously throughout the pandemic and was composed of clinical leaders representing a range of patient care disciplines, including inpatient and ambulatory patient care, emergency department, hematology/oncology, infectious diseases, occupational health, laboratory medicine and pathology, surgery, and infection prevention.

**TABLE 2:** Clinical testing scenarios and associated service level agreements including turnaround time, location of testing performance, and potential to sending out to a reference laboratory^*c*^

Clinical scenario	Target TAT (hrs)	Local or central testing^*a*^	Send-out allowed	Triage method
ED and inpatient setting				
Priority	2	Local	No	Label icon + “runner”/hand delivery
Non-priority	6	Local	No	
Ambulatory setting (includes ambulatory collection sites, draw stations, and provider offices)				
Pre-procedure testing	24	Local	No	Label icon + courier delivery
Healthcare workers (HCW)	24^*b*^	Local	No	
Symptomatic	24^*b*^	Central	No	
Asymptomatic	48^*b*^	Central	Yes	
Contracted clients	48	Central	Yes	Courier delivery

^*a*^ Local or centralized testing practices changed over the course of the pandemic depending on supply chain constraints and workflow efficiencies. The testing location indicated here was the scheme used for most of the study period.

^*b*^ TAT targets varied throughout the pandemic—HCW 24–36; ambulatory symptomatic 24–36; ambulatory asymptomatic 48–72. The target that was used for the majority of time during the pandemic is shown in the above table.

^*c*^ Abbreviations: ED, emergency department; TAT, turnaround time; HCW, health care worker.

### Ask at order entry questions

**Fig 1:**
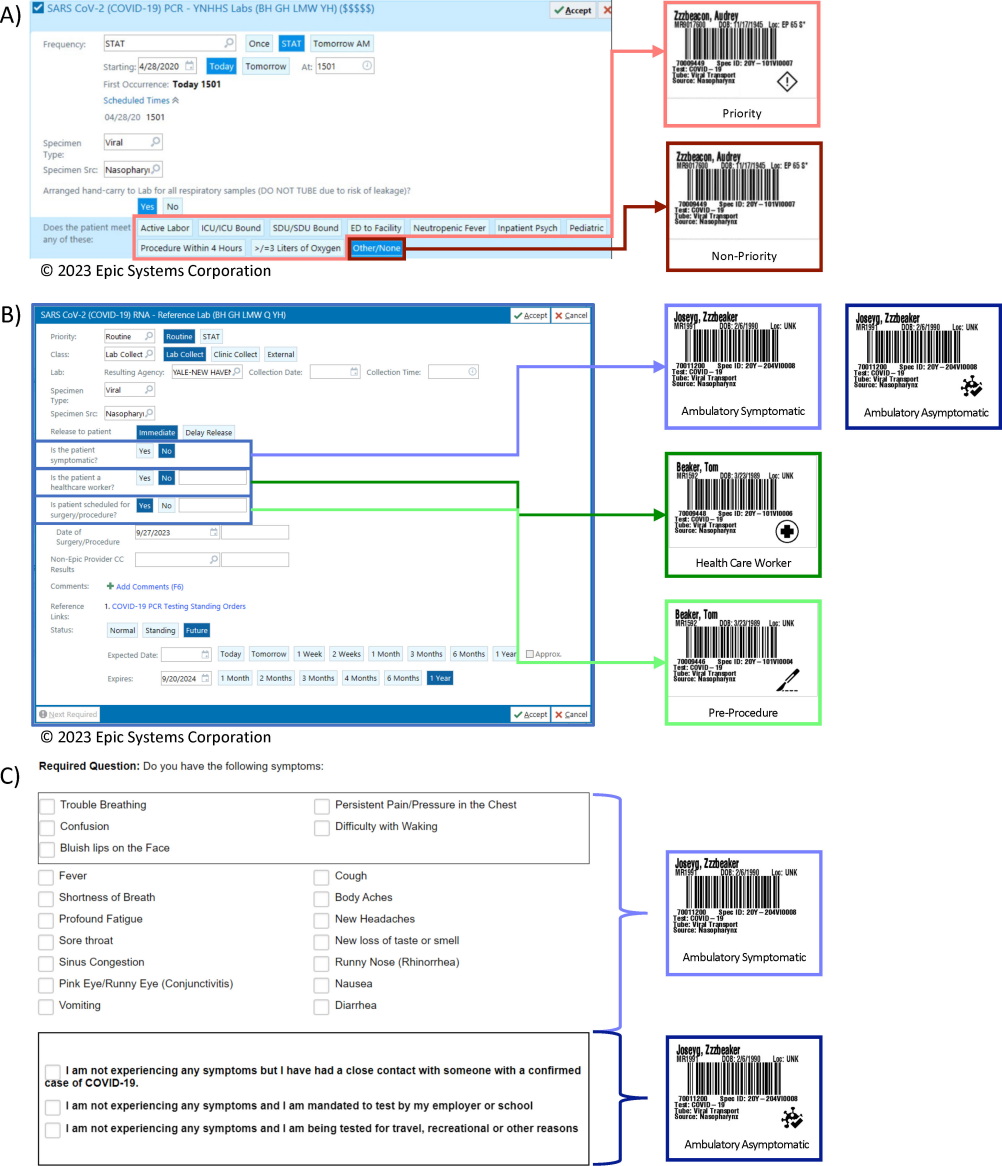
AOE questions embedded within the (**A**) inpatient and emergency department and (**B**) ambulatory order screens and (**C**) patient-facing website for SARS-CoV-2 testing and relationship to label icon placement. A restricted healthcare worker-facing website is not shown. AOE responses directly drove icon placement for providers using the computerized order entry system in both inpatient and ambulatory settings. Website responses created distinct ambulatory visit types that drove icon placement. Specific criteria and questions may have changed over the course of the pandemic; these are representative of the AOE questions and format. (panels A and B 2023 Epic Systems Corporation.)

AOE questions were presented to providers when ordering SARS-CoV-2 NAAT via CPOE in the ED or inpatient setting (Fig. 1A). These questions were developed by the COVID-19 Testing Stewardship Committee such that responses indicated the relevant clinical scenario that prompted testing. The discrete data elements collected from these AOE questions drove downstream within-laboratory CDS via specimen label icons (described below).

In the ambulatory setting, AOE questions were presented to providers using CPOE including procedure-based specialties ordering pre-procedure testing (Fig. 1B). For patients and healthcare workers initiating testing independent of a healthcare visit, we created community-facing and password-restricted websites that incorporated AOE questions (Fig. 1C).

### Specimen label icons

For inpatient and ED care areas and ambulatory sites using the CPOE, responses to AOE questions directly drove the printed label icon. For community and HCW websites, AOE question responses created visit types associated with label icons (Fig. 1A through C). Compatible label printers were available at patient care areas, and custom programing through the Zebra Printing Language was used to print the appropriate icon on specimen labels at the time of collection.

### Specimen transport and triaging

**Fig 2:**
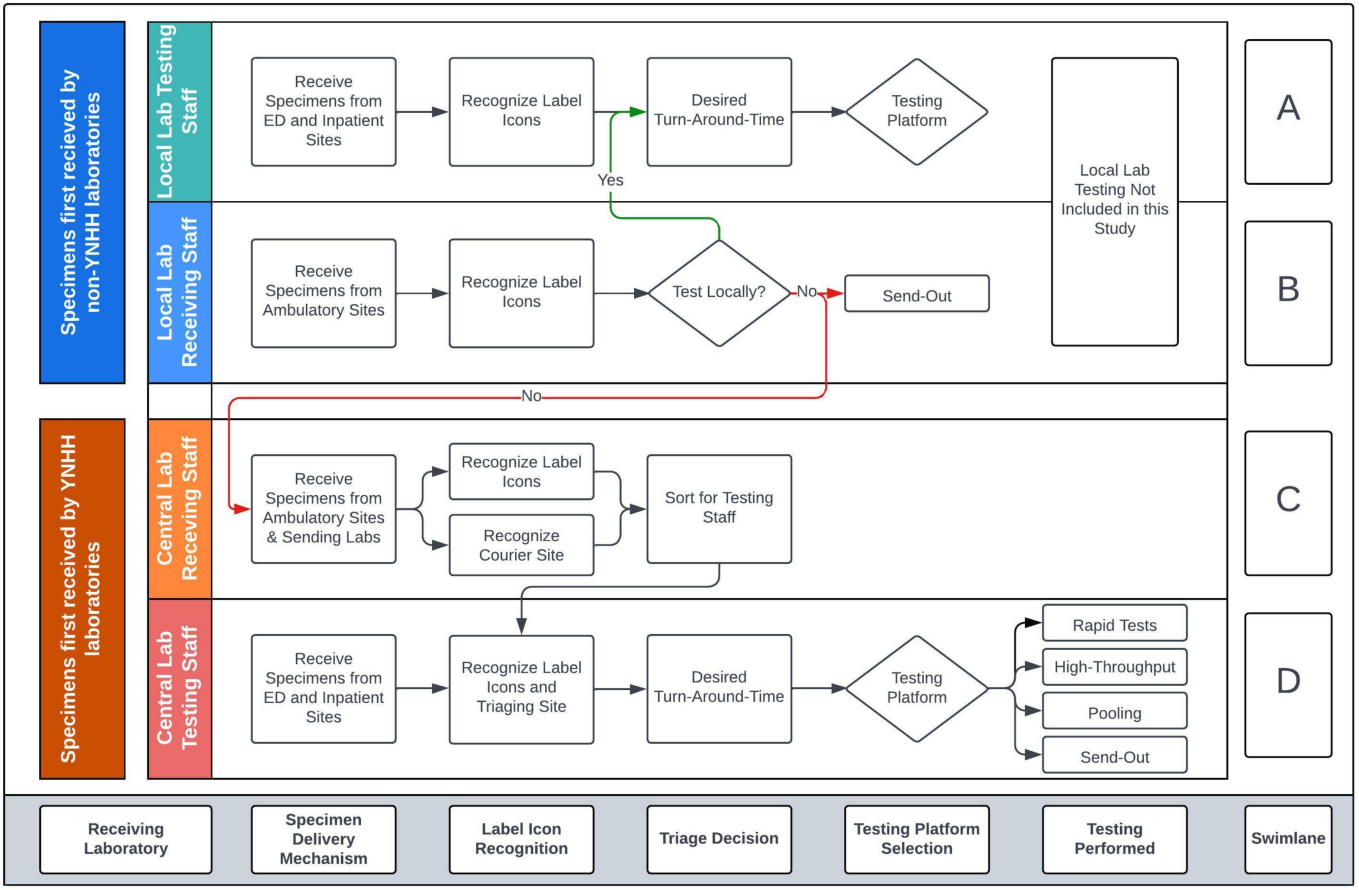
Specimen triaging by local laboratories, central receiving staff, and laboratory testing staff using couriers and icons. Samples sent directly to YNHH from collection sites, those sent from contract clients, or those sent from other health system facilities for internal reference testing were received and triaged by dedicated COVID-19 specimen receiving staff (Swimlane C). ED and inpatient testing was directly received in the testing laboratory (Swimlane D). Only testing performed at YNHH laboratories was included in the analysis. The bottom of the panel indicates the essential steps in the triage workflow.

Within- and between-laboratory workflows with triage points, including those incorporating label icons, are shown in the Swimlane diagram in Fig. 2. Swimlane diagrams are used to outline workflow processes involving different work areas to delineate actions and responsibilities. For specimens collected at other facilities within our health system, including ambulatory collection sites, initial receipt and triaging occurred at the local laboratory (Fig. 2, Swimlanes A and B). Samples from health system sites that were not sent to YNHH for internal reference testing were excluded from analysis. Specimens originating at other facilities in our health system were also sent to YNHH for internal reference testing (Fig. 2, Swimlanes B to C)

Within the YNHH laboratory, ED and inpatient obtained specimens were hand delivered to the laboratory, and triaging was based on label icon and delivery method (Fig. 2, Swimlane D). Specimens from all ambulatory clinical scenarios from YNHH-administered ambulatory collection sites were delivered *en bulk* to common specimen receiving areas. This included up to seven tent-based collection sites and over 20 draw stations (Fig. 2, Swimlane C). For both ED/Inpatient (Fig. 2, Swimlane D) and ambulatory workflows (Fig. 2, Swimlane C), some specimens had no label icon, and the absence of an icon in conjunction with the delivery mechanism served as the CDS triage tool. For ED/Inpatient or ambulatory settings, no icons corresponded to non-priority or symptomatic specimens, respectively. Receiving staff used courier and label icon information to sort specimens for testing staff who then used this information to choose among available testing platforms to meet desired TAT (Table 2; Fig. 2).

### Data extraction

We extracted specimen-level data from several sources including the LIS, localized COVID-19 testing dashboards, and a common laboratory data repository. All data extracts were integrated using the unique specimen identification number, thereby aggregating all the requisite information into a single data file for analysis.

### Data analysis

Information for “Diamond” (priority ED/inpatient) and “Scalpel” (pre-procedure) icon generation was explicitly stored in the LIS and linked to specimens. For other clinical scenarios, we retrospectively categorized specimens based on extracted information and logic in use at the time of sample collection using a custom Python (v3.9.15) script. The following clinical scenarios and label icons were combined due to comparable clinical scenarios and identical TAT targets: ED and inpatient combined SARS-CoV-2, influenza A, influenza B, and respiratory syncytial virus testing were considered “priority,” and ambulatory orders for combined SARS-CoV-2, influenza A, and influenza B were considered “Symptomatic.”

All samples collected under the umbrella of Yale New Haven Health had discrete collection and verification times within the LIS. Sample TAT was the difference between collection time and verification time in hours. External samples may have had incomplete or incorrect collection times and were excluded from TAT analysis.

Population medians and interquartile ranges and confidence intervals with Bonferroni correction for 104 weeks (99.95% confidence intervals) were calculated. Confidence intervals around the median for each MMWR week for each clinical scenario were calculated by bootstrapping method using 1,000 samples with replacement using custom Python scrips. All statistical analyses were implemented in Python (v3.9.15) with NumPy (v1.23.5) and SciPy (v1.9.3). Lastly, two-way Kruskall-Wallace and post-hoc tests were performed in GraphPad Prism (v9.4.1).

We analyzed the TAT for SARS-CoV-2 NAAT testing across patient care settings (e.g., inpatient/ED and ambulatory). We also performed a sub-group analysis for different clinical scenarios set by the testing committee, such as symptomatic/asymptomatic patients, pre-procedure cases, and healthcare workers, taking into account changes in test volume.

## RESULTS

### Overall testing patterns

**Fig 3:**
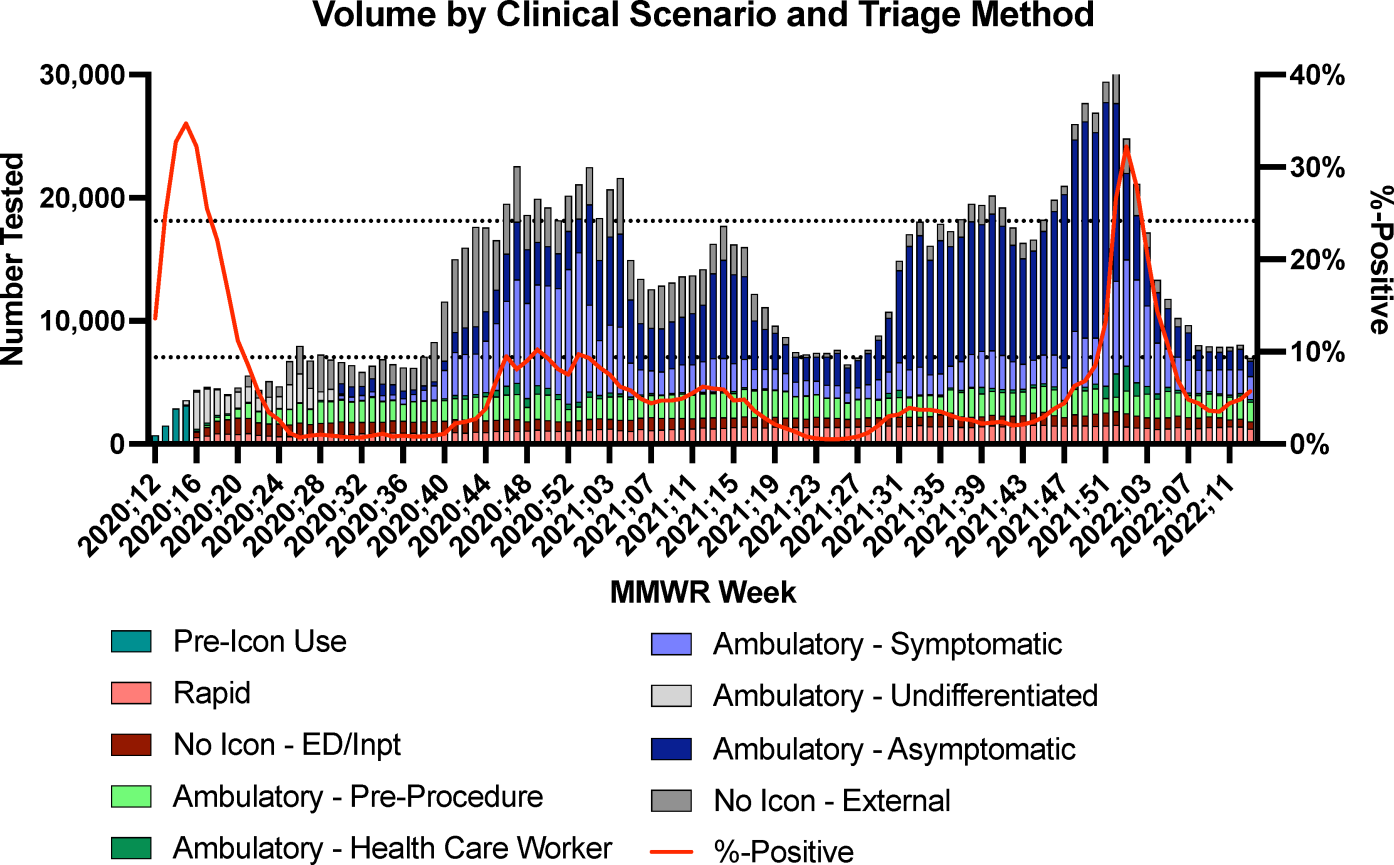
Testing volume by MMWR week and clinical scenario and triage mechanism and overall %-positivity for each week of testing. Horizontal lines indicate 25th and 75th percentiles for total testing volume.

Our health system began testing for SAR-CoV-2 in March 2020. Label icons and unique visit types were implemented in April 2020 with categories being added and/or label icon logic being modified throughout the pandemic. Between March 2020 and June 2022, we collected and/or performed 1.39 million COVID-19 tests across all patient categories (Fig. 3). Testing for ED, inpatient, and pre-procedure populations were stable over the pandemic, while HCW and ambulatory testing fluctuated substantially depending on the rates of community transmission and demand for testing. Among included specimens, 57.3% of specimens had a label icon. For inpatient and ED specimens, 57.6% had a label icon, and 69.4% of ambulatory specimens directly collected by our health system had a label icon. Among the 42.7% of specimens lacking a label icon, approximately one-third were collected in environments outside our health system (labeled “External” in Fig. 3); these were most often nursing homes and differentiated by courier drop offs.

### ED and inpatient testing

**Fig 4:**
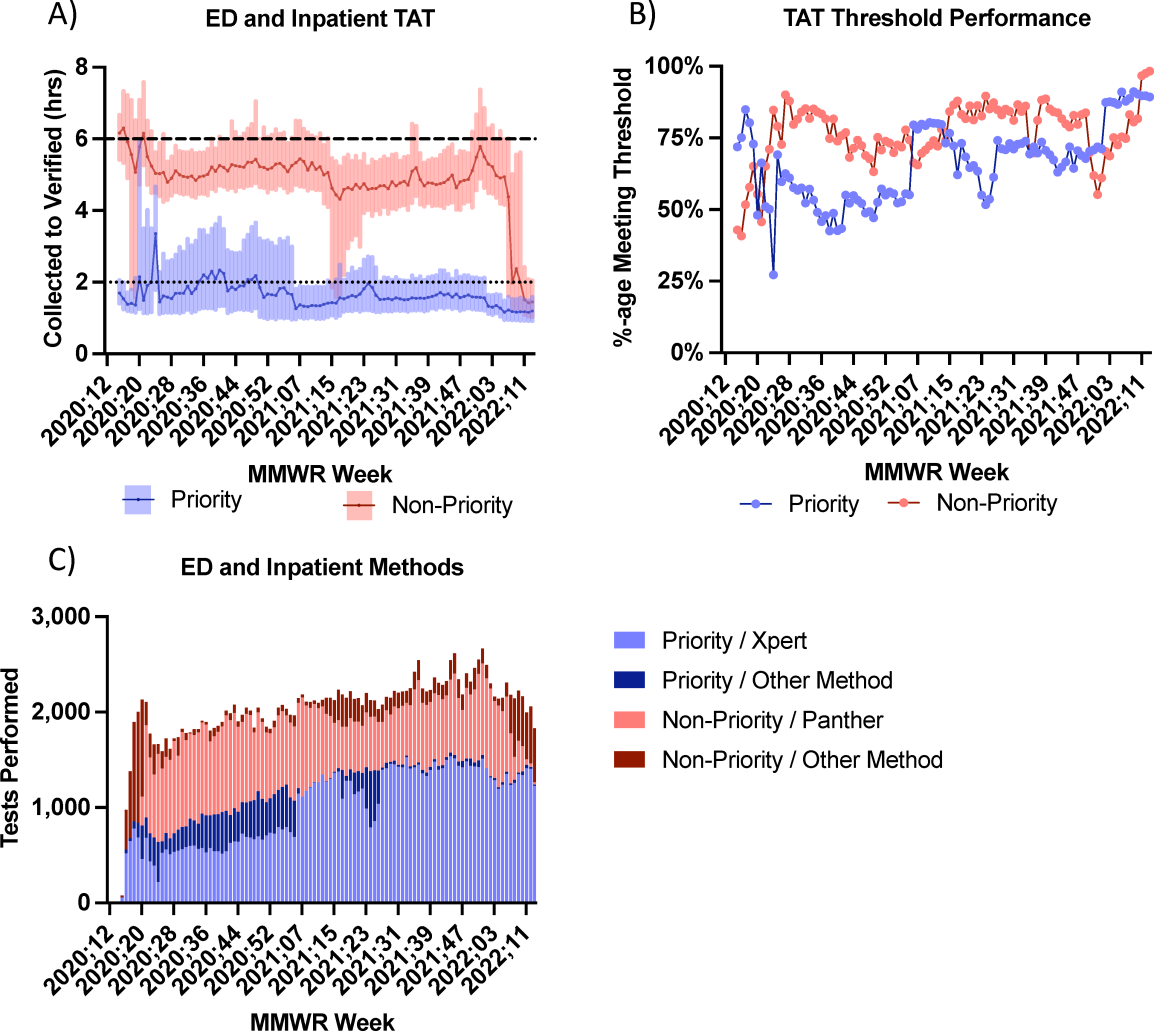
TAT (**A**), TAT threshold performance (**B**), and volume by testing method (**C**) for ED and inpatient care areas. TATs are shown in hour from the time of collection to the time of verification. (**A**) Line is median, and shaded areas are interquartile ranges. Horizontal dotted line at 2 hours indicates TAT target for priority specimens, and horizontal dashed line at 6 hours indicates TAT target for non-priority specimens. (**B**) The percentage of priority and non-priority specimens meeting the desired TAT thresholds are shown. (**C**) Priority specimens tested by Cepheid Xpert methods versus other methods and non-priority specimens tested by Hologic Panther versus other methods used by MMWR week are shown.

All testing from ED and inpatient areas was ordered within EPIC, our CPOE system, and interaction with AOE questions determined “Diamond” icon placement indicating priority or non-priority status (Fig. 1A). Specimens were hand carried and received into the testing laboratory, and label icons were recognized in conjunction with delivery mechanism for specimen triaging and test platform selection (Fig. 2, Swimlane D). Among ED and inpatient populations, SARS-CoV-2 NAAT median TAT significantly differed across all testing weeks between priority and non-priority specimens (Fig. 4A; Fig. S1). The median TAT for priority specimens was consistently below the 2-hour target, but the percentage of specimens meeting the 2-hour threshold varied (Fig. 4B). A higher percentage of non-priority specimens met their 6-hour TAT target. Interestingly, the period of lowest TAT compliance for priority specimens, MMWR weeks 2020.35 to 2021.05, was not associated with the highest sample volume, but TAT was delayed due to reagent constraints secondary to manufacturer allocations leading to use of alternative testing platforms (Fig. 4C). As reagent constraints eased in MMWR week 2022.08, there was a transition to more rapid testing platforms for all ED and inpatient specimens to simplify laboratory workflow. (Fig. 4C).

### Ambulatory patient population testing

In the ambulatory setting, there were four patient testing clinical scenarios: (1) asymptomatic (2), symptomatic (3), pre-procedure, and (4) HCW. Label icons were assigned based on AOE question responses within the CPOE system or a patient- or HCW-facing website (Fig. 1B and C), and triaging decisions were made by the receiving laboratory based upon the label icon (or its absence) in conjunction with its delivery mechanism (Fig. 2).

**Fig 5:**
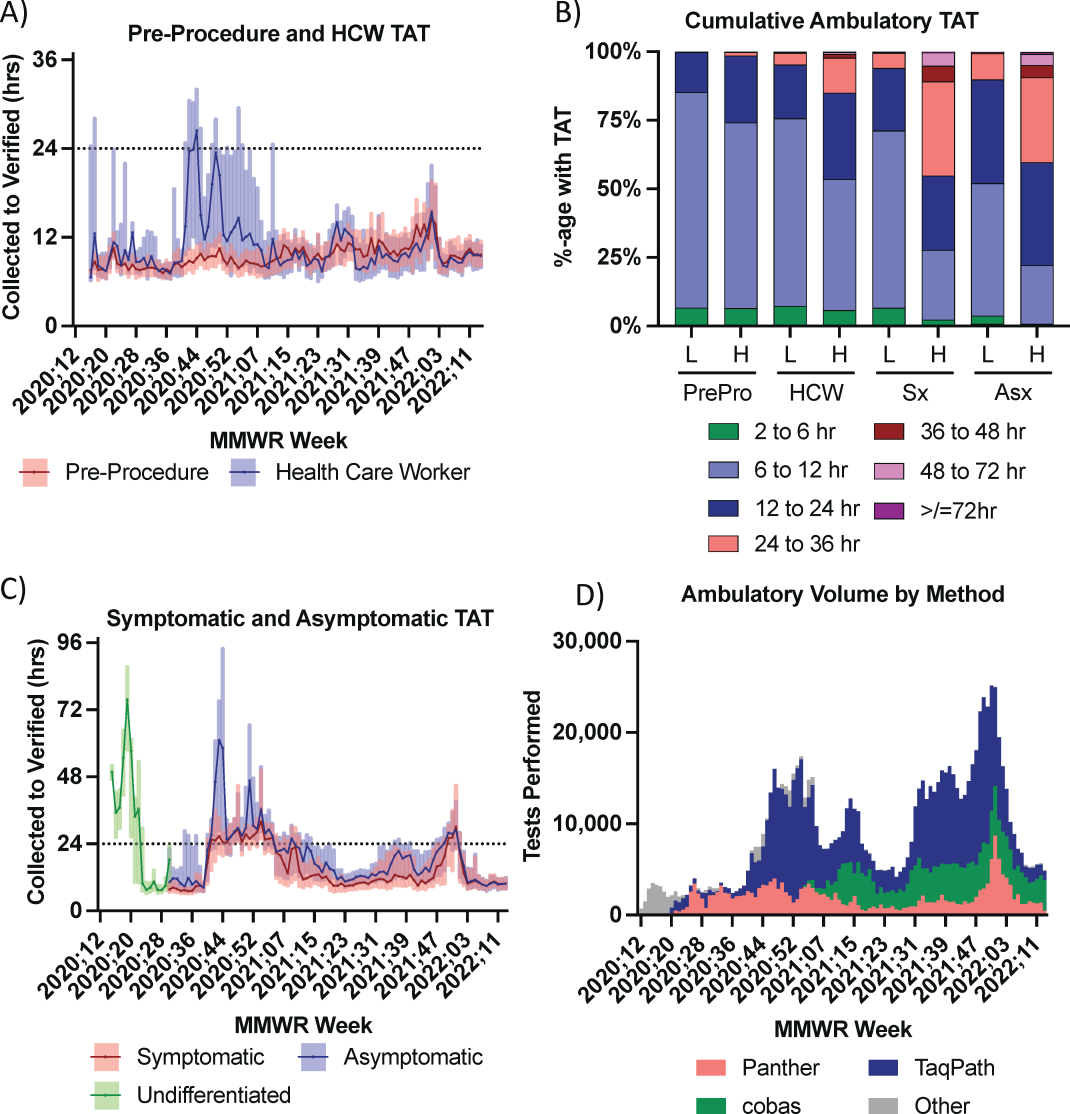
Turn-around-time (TAT) (A and C), cumulative TAT performance (B), and volume by testing method (C) for ambulatory testing clinical scenarios. (A) TAT in hours from the time of collection to the time of verification for pre-procedure and health care worker testing. Lines are median, and shaded areas are interquartile ranges. (B) The percentage of specimens for pre-procedure (PrePro), HCW, symptomatic (Sx), and asymptomatic (Asx) ambulatory patients with the TAT within each time frame is shown for samples collected in weeks with total testing volume in the top 50%-ile [high (H) volume] or lowest 50%-ile [low (L) volume]. (C) TAT in hours from time of collection to time of verification for symptomatic and asymptomatic ambulatory patients. Lines are median, and shaded areas are interquartile ranges. Undifferentiated indicates non-pre-procedure, non-HCW ambulatory testing before implementation of asymptomatic label icons. (D) Testing platform utilization among ambulatory patients for the indicated MMWR week is shown.

The target TAT for pre-procedure and HCW testing across our health system was 24 hours, and most pre-procedure and HCW testing was performed at the laboratory affiliated with the sample collection location as shown in Fig. 2, Swimlane B to Swimlane A for samples collected at non-YNHH sites or Swimlane C to Swimlane D for samples collected at YNHH sites. The median TAT for pre-procedure testing was below 12 hours for nearly all weeks (Fig. 5A). TATs greater than 12 hours with wider interquartile ranges were seen during the Omicron surge in the winter of 2021 and 2022. Collectively, over 98% of pre-procedure samples had TAT less than 24 hours under both low- and high-volume testing periods (Fig. 5B).

Median TAT for HCW testing was largely in range with the target TAT of 24 hours when supplies and workflow allowed local testing to occur (Fig. 5A). However, substantial fractions of HCW testing were sent to YNHH (Fig. 2, Swimlane B to C) for internal reference testing from MMWR weeks 2020.40 to 2021.07 due to reagent constraints secondary to allocations, and this was associated with prolonged and greater variation in TAT reflecting additional processing and courier time (Data not shown). There was also an increase in TAT for HCW testing associated with the onset of the Omicron surge (MMWR week 2021.50). Over 75% of all HCW testing met the target TAT of 24 hours (Fig. 5B), but compliance was lower during periods of high test volume. Median TAT significantly differed for pre-procedure and HCW samples in 43 out of 104 weeks of testing, with most differences seen during periods of high testing volume (Fig. S1).

The target TAT for symptomatic and asymptomatic ambulatory populations varied, with desired symptomatic TAT ranging from 24 to 36 hours and asymptomatic target TAT < 48 hours with some periods of higher TAT targets. These thresholds were predominantly met with the greatest excursions during periods of high demand (Fig. 5C). Based on the analysis of the 99.95% confidence intervals for the 89 weeks when symptomatic and asymptomatic samples were differentiated, median TATs were significantly different in 69 out of 89 weeks (Fig. S1). Of the 20 weeks with comparable median TAT, 10 occurred after a laboratory process change in MMWR week 2022.04 led to substantially reduced TAT for all ambulatory clinical scenarios.

Ambulatory testing across all clinical scenarios was primarily performed using Hologic Panther, Roche cobas, or ThermoFisher TaqPath test systems (Fig. 5D). Panther and cobas test systems were predominantly used for pre-procedure and HCW testing with more stringent TAT targets. The TaqPath assay was important for higher volume testing of symptomatic and asymptomatic samples, especially during periods of high demand, despite more challenging workflow requirements. Within laboratory CDS strategies drove platform selection to meet TAT targets.

### Impact of label icons on ambulatory testing clinical scenarios

**Fig 6:**
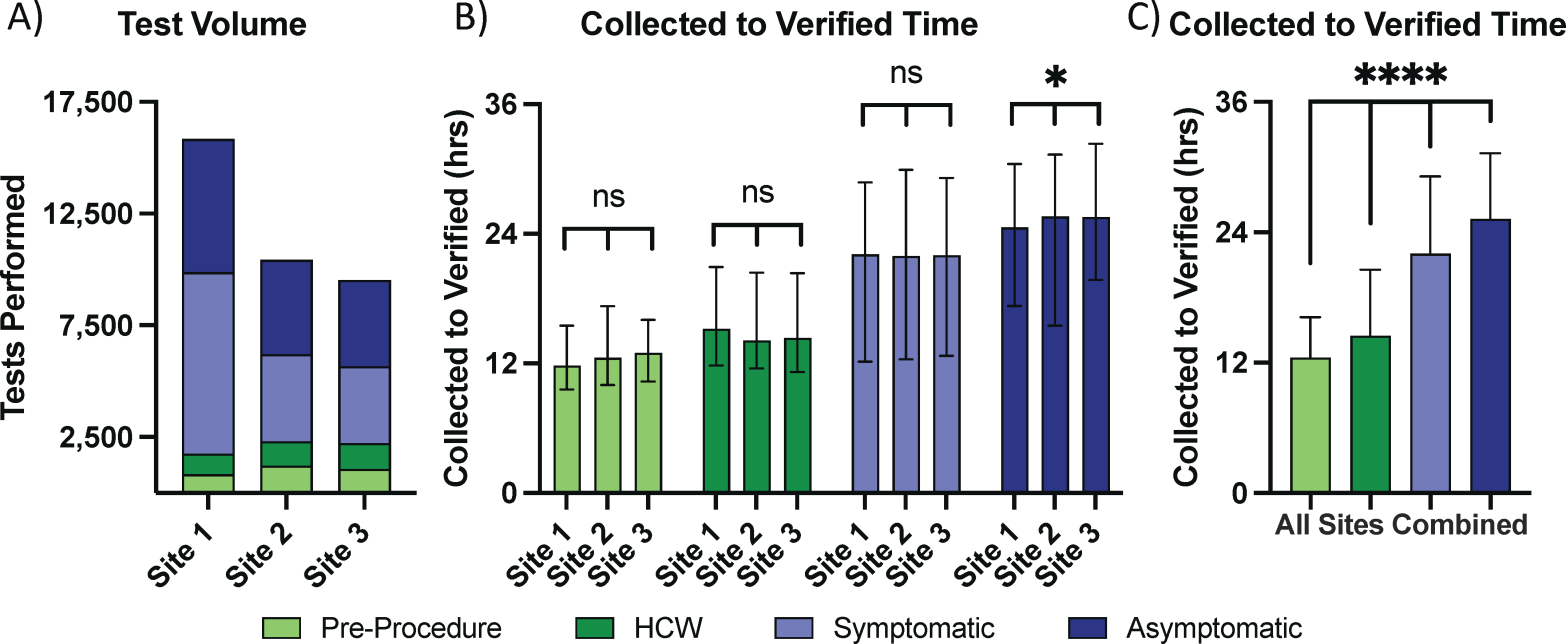
Testing volume (**A**) and turnaround time (**B, C**) for three ambulatory collection sites by clinical scenario for MMWR weeks 2021.51 to 2022.02. All sites collected pre-procedure, HCW, symptomatic, and asymptomatic patients 7 days a week throughout the day. All sites had four direct couriers per day to the central testing laboratory. TAT is shown as median and interquartile range. (**B**) Two-way Kruskall-Wallace testing was performed with post-hoc comparisons with Dunn’s correction. TAT was significantly different (*P* < 0.0001). Both clinical scenario (*P* < 0.0001) and collection site (*P* = 0.0386) were significantly associated with differences in TAT. Within clinical scenarios, there were not significant differences among collection sites for samples from pre-procedure, HCW, and symptomatic patients. There was a significant difference among sites for specimens from asymptomatic patients (Site 1 vs Site 2, *P* = 0.0108, and Site 1 vs Site 3, *P* < 0.0001). (**C**) TAT for all collection sites together are shown. TAT was significantly different among all clinical scenarios by Kruskall-Wallace test and post-hoc comparisons with Dunn’s correction (overall *P* < 0.0001; all post-hoc comparisons, *P* < 0.0001).

To better demonstrate the impact of label icons for within-laboratory CDS, we analyzed a subset of specimens collected at three ambulatory collection sites during 4 weeks from 2021.51 to 2022.02. These collection weeks were selected because they encompassed the highest testing volume, highest symptomatic testing volume, and highest positivity rate when widespread testing was available (Fig. 3). During this time, these sites collected ~35,000 specimens, corresponding to ~1/3 of all specimens tested. The included sites collected a high volume of pre-procedure, HCW, symptomatic, and asymptomatic samples throughout the day across all days (Fig. 6A and data not shown). All three sites sent specimens directly to the YNHH laboratory via multiple courier runs each day. Two-way Kruskall-Wallace testing with Dunn’s correction demonstrated significant differences in TAT for each clinical scenario (*P* < 0.0001) and collection site (*P* = 0.0386) (Fig. 6B). Post-hoc testing revealed no significant differences among all three collection sites for pre-procedure, HCW, and symptomatic testing, but TAT for sites 1 and 2 and 1 and 3 differed significantly for asymptomatic testing. When data from all collection sites were combined, TAT significantly differed among all clinical scenarios (Fig. 6C).

## DISCUSSION

Traditional specimen triage methods were not sufficiently scalable or adaptable to meet the clinical needs for SARS-CoV-2 NAAT at our institution. New specimen triaging pathways were needed to address diverse patient groups with varying TAT and processing needs, often originating from identical ambulatory collection sites. In the present study, we describe a novel approach for within-laboratory CDS using AOE question-driven specimen label icons to facilitate specimen triaging to achieve institutional goals for TAT. This system was implemented using existing EHR and LIS resources within a health system without a history of high-volume reference testing.

Designing effective CDS systems can be challenging, but current literature offers a pathway for the CDS development process. The “Ten Commandments for Effective Clinical Decision Support” and the “5 Rights of Clinical Decision Support” are two prominent examples that can be used in healthcare (7, 11). The guidelines in the “Ten Commandments” of CDS include ensuring that CDS aligns with clinical workflows, is evidence based, is integrated with other health information technology systems, is usable and intuitive for clinicians, and is regularly evaluated and updated (7). The “5 Rights” include the right information, right format, right channel, right time, and right intervention (11). These frameworks collectively seek to ensure that CDS systems are effective, safe, and well received by end-users.

We highlight that not all decision support alerts should be electronic when implementing CDS systems, especially within the laboratory (11). During triaging at our institution, clinical specimens were separated for designated workflows without the need to interact with the LIS using visual cues. We considered the use of electronic icons displayed on packing lists as potential solution, but staff felt that a visual aid on the physical specimen would permit faster decision making. Color-coding specimens or bags was not thought to be adequately scalable, and triage information would not be maintained within the LIS. Additionally, colored bags were difficult to obtain due to supply chain constraints. Taken together, the specimen label icon aligns with the CDS Five Rights and the Ten Commandments of CDS Development in several ways but specifically, in that they present the information to the right person, at the right time during a workflow, and permit quick decision making.

The effectiveness of AOE questions is dependent upon providers answering questions honestly, and there were concerns that providers were being dishonest with their responses to obtain testing faster than otherwise indicated. However, AOE question responses were discrete, objective, and verifiable allowing intermittent audits that did not reveal widespread problems. When erroneous responses to AOE questions were found, feedback was given to providers and departmental leadership. Compliance with AOE questions was likely related to the multi-disciplinary institutional stewardship committee responsible for assigning priorities to testing. Inpatient and ED leaderships, including at the department chair level, were active participants in the committee lending credibility to the priorities and guidelines developed for front line staff.

Within the institution, all clinical services used the same orders for SARS-CoV-2 NAAT throughout the pandemic. There were no differences among inpatient care areas for the testing performed. As structured, the AOE questions did not require substantially more provider interaction than other orders, and there was no notable resistance to the use of AOE questions. Requests for expanded indications for priority testing were made to the COVID-19 Testing Stewardship Committee, but no requests for exemption from AOE questions were made. Alternative CDS tools (e.g., duplicate test stops) were used when providers tried to order testing under some circumstances, but those interventions occurred prior to samples reaching the laboratory.

The AOE question paradigm offered benefits over traditional specimen triaging frameworks used by laboratories (e.g., “STAT” labels). The COVID-19 Testing Stewardship Committee noted that the use of STAT is subjective is potentially susceptible to misuse, and offers limited resolution due to its binomial nature (STAT and non-STAT), particularly when monitoring TAT targets. In contrast, AOE questions afford a higher resolution for auditing the TAT for specific clinical scenarios. This granular approach enabled the COVID-19 Testing Stewardship Committee to monitor TAT dashboards throughout the pandemic and revise specimen collection and triaging practices as required. Additionally, while not formally studied, the laboratory staff provided very positive feedback on the use of label icons, and new icons were deployed throughout the pandemic, at the request of testing staff, as new testing options and clinical scenarios were added.

Secondary advantages of the AOE question approach lie in its configurability and permanence within the LIS or medical record. We were able to rapidly modify indications for priority testing in inpatient and ED settings based on the availability of tests and emergent needs without necessitating retraining of clinical and laboratory staff. The underlying icon information persisted within the LIS and was available on outstanding list review including samples sent for internal reference testing. Reprinted labels also contained correct icon information enabling downstream workflows. This contrasts with other triage methods, including color-coding or physical notes in bags where specimen-specific triage information may be lost. The permanence of this information within the LIS and EHR facilitated laboratory and quality dashboards used throughout the pandemic.

A substantial amount of testing occurred on the ThermoFisher TaqPath assay, and this assay was extremely challenging to implement leading to variations in practice over the course of the pandemic. Due to concerns for spurious single-gene detections, the risk of carry-over among adjacent wells, and challenges with sample mixing, we routinely performed repeat testing on samples testing positive on the TaqPath assay (12, 13). However, different repeat testing protocols were used at different times, leading to variations in TAT especially for positive samples. This was pronounced during the winter of 2021–2022 when positivity rates exceeding 30% were seen among symptomatic patients. This prolonged TAT for the symptomatic patient population, and repeat testing was preferentially performed on Panther and cobas instrumentation reducing the capacity on these more straightforward testing platforms. When repeat testing criteria were relaxed in early 2022, TAT for all ambulatory populations fell substantially, and this was related to more immediate verification and release of strongly positive samples tested by the TaqPath assay as well as shifting more primary patient testing to the Panther and cobas systems.

As an institution, we early on elected to use “collected to verified” TAT to capture the total testing process instead of “received to verified,” which predominantly reflects laboratory practices. This decision was made because (1) our health system was responsible for all aspects of test ordering, specimen collection, and result generation, and we wanted to reflect the totality of all processes, (2) during periods of peak sample volume, there may have been delays within the laboratory between physical receipt of the specimen and its receipt into the LIS potentially giving erroneously short TAT, and (3) our goal to was to reduce global TAT for patient and provider satisfaction, and collected-to-verified was felt to be the best available metric for this. For reference laboratories or those with less control over pre-analytic processes, “received to verified” may be a more appropriate measure, but the use of within-laboratory CDS via label icons would be expected to provide similar triage benefits in these cases.

This study has several limitations. The laboratory could not conduct a formal workflow analysis of the label-icon process during the intense demands of the pandemic, and this approach often forms a critical part of process redesign. Additionally, we implemented the label icon system very early in the pandemic, and we could not compare icon usage to no icon usage meaningfully. The integration of usability testing could have provided insights into the learnability and memorability of the new workflow. Moreover, we focused on laboratory metrics associated with the icon-based within-laboratory CDS, but we did not evaluate the impact on patient outcomes. As CDS research continues to advance, future investigations need to incorporate patient outcomes into their study designs. Additionally, the logic leading to placement of some icons was complex and changed (e.g., HCWs), and our retrospective label assignment may have misclassified some of these samples. Lastly, inconsistencies in label printing processes and reliance upon courier drop-off routines, especially for samples received from facilities outside our health system, could have resulted in specimens missing appropriate icons and/or being mis-triaged.

### Conclusion

The AOE question-response paradigm and within-laboratory CDS using specimen label icons substantially enhanced our laboratory’s ability to efficiently triage specimens. This methodology facilitated the achievement of our TAT targets for different clinical scenarios by streamlining the specimen testing process across various platforms within the laboratory. This approach’s effectiveness was particularly pronounced among the highest priority specimens during periods of elevated sample volumes, showing the utility and scalability of this novel triaging strategy in times of heightened demand.

## SUPPLEMENTAL MATERIAL

Figure S1 jcm.00785-23-s0001.pdf Median and 99.95% confidence intervals.

ASM does not own the copyrights to Supplemental Material that may be linked to, or accessed through, an article. The authors have granted ASM a non-exclusive, world-wide license to publish the Supplemental Material files. Please contact the corresponding author directly for reuse.
