# Supplementary material for: Retrospective evaluation of clinical decision support for within-laboratory optimization of SARS-CoV-2 NAAT workflow
Source: J Clin Microbiol. 2023 Dec 22;62(2):e00785-23. doi: 10.1128/jcm.00785-23 (PMC10865785; doi:10.1128/jcm.00785-23)
Supplement: Figure S1 — Median and 99.95% confidence intervals. [file jcm.00785-23-s0001.pdf]

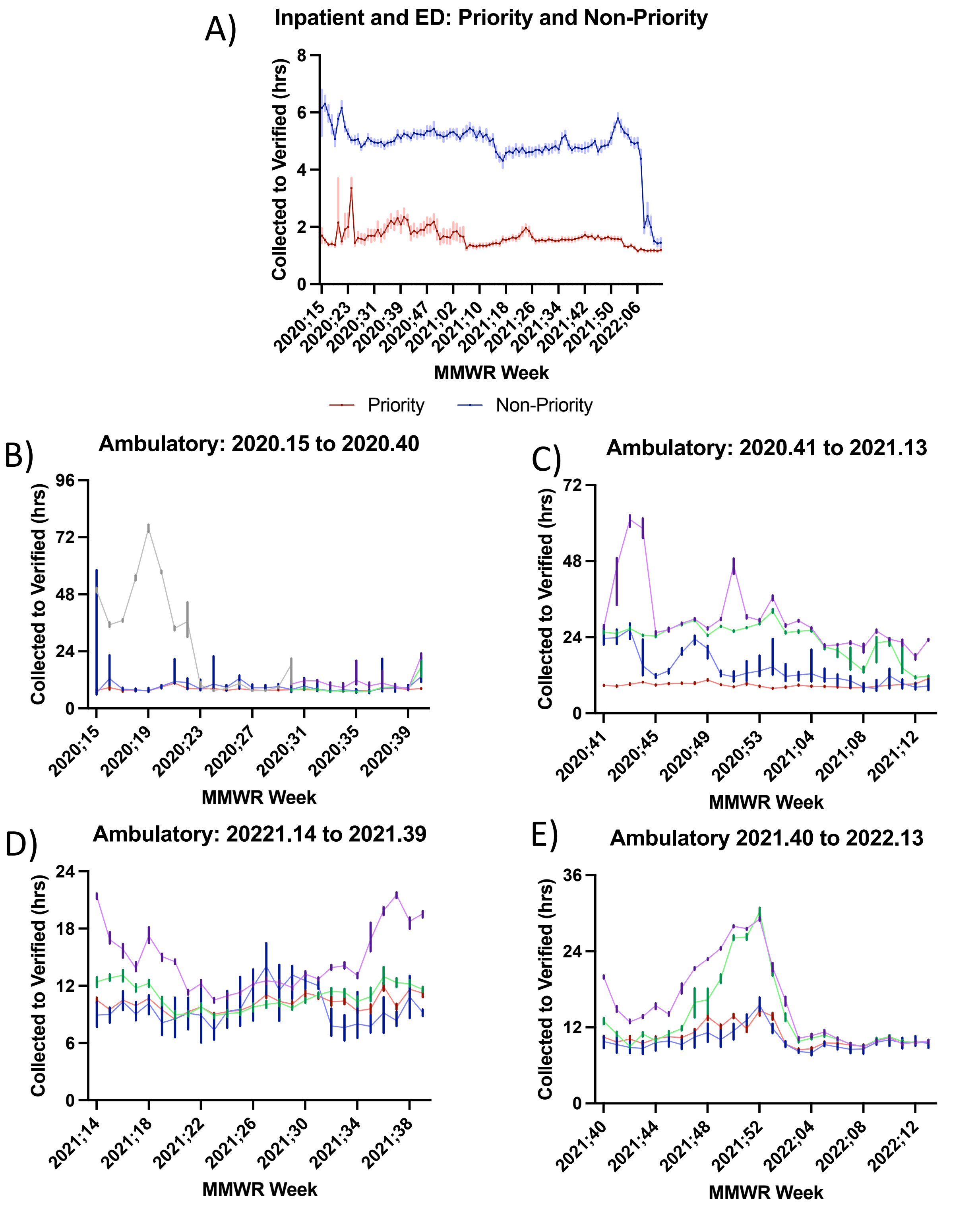

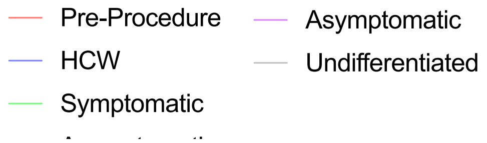

Supplemental Figure 1: Median turn-around-time and 99.95% confidence intervals for A) Inpatient and ED and B) to E) ambulatory clinical scenarios. Confidence intervals were calculated by bootstrapping 1,000 samples from the week and clinical scenario. For B) to E) y-axis values differ among charts due to variation median TAT.
